# Family Medicine at the Forefront: Lessons Learnt From the COVID-19 Vaccine Rollout in Crete, Greece

**DOI:** 10.3389/fpubh.2022.815825

**Published:** 2022-01-31

**Authors:** Christos Lionis, Marilena Anastasaki, Elena Petelos, Kyriakos Souliotis, Ioanna Tsiligianni

**Affiliations:** ^1^Clinic of Social and Family Medicine, Faculty of Medicine, University of Crete, Heraklion, Greece; ^2^Health Services Research, Faculty of Health, Medicine and Life Sciences, CAPHRI Care and Public Health Research Institute, Maastricht University, Maastricht, Netherlands; ^3^Faculty of Social and Political Sciences, University of Peloponnese, Corinth, Greece; ^4^Health Policy Institute, Athens, Greece

**Keywords:** vaccination, COVID-19, primary care, family medicine, Greece

## Introduction

With the world being amidst the global coronavirus pandemic, the development of vaccines to prevent further transmission was urgently needed, and the response to address this need has been unprecedented. On 21 December 2020, the European Commission (EC), upon a positive scientific recommendation of the European Medicines Agency (EMA), granted the first Conditional Marketing Authorization (CMA) to a vaccine against SARS-CoV-2 (Comirnaty of BioNTech and Pfizer) (1). The CMA was granted for preventing coronavirus disease 2019 (COVID-19) in people from 16 years of age.

On December 27, the COVID-19 vaccination rollouts started across the European Union (EU) Member-States with healthcare and other frontline workers; older adults were identified as priority groups across jurisdictions, while different priority groups were established across different countries. In Greece, January 16 was the first day for the vaccine administration to adults from 85 years of age. Greece introduced a staggered vaccination rollout based on age and exposures. The University Hospital of Heraklion (PAGNI) in Crete was amongst the first healthcare units to participate in this effort. The task was assigned to the PAGNI's Department of Public Health, under the coordination of three university professors and with the participation of 17 residents trained in General Practice/Family Medicine (GP/FM).

This perspective article aims to snapshot observations and experiences gathered from the COVID-19 Vaccination Centre (VC) established at the University Hospital of Heraklion in Crete. We also seek to discuss how key practice aspects could be used to inform future interventions and to translate the experiences gained into concrete proposals to improve the role of healthcare practitioners in GP/FM and in primary health care (PHC), to improve the efficiency and effectiveness of future rollouts, as well as to combat similar crises in the future. Finally, We share the first experiences and observations made to date. Furthermore, we formulate our experiences into recommendations aiming to inform the transformation PHC urgently requires in Greece needs to enhance the resilience of the healthcare system, increase preparedness, and improve response.

## Experiences Gained

To date (October 1st, 2021), adults, and children from 13 years of age, have been presenting for vaccination to the VC of PAGNI in Crete. During the pre-vaccination consultation, a brief medical history, regarding comorbidities and medications was obtained, along with information on recent episodes of flu or other infections, recent vaccination uptake, and history of contact with any confirmed or suspected COVID-19 case. The first and last author have involved in the providing the services at the VC and supervised the whole process upon the endorsement of the administrative authorities of the University Hospital. Although, it is an audit paper that it has been written as an opinion, the Hospital leaders have fully approved it.

To facilitate the flow of the information in this report, we classified the experiences gained from the VC in three time slots and specifically:

- the first one corresponds to the first 4 months (January–April 2021), i.e., the vaccination of older adults;
- the second (after April 2021–now) corresponds to the period of the first adverse reactions and hesitancy emerging and resulting in a sustained effect against the COVID-19 vaccination attributed to the reported adverse reactions of Vaxzevria (Astra Zeneca);
- the third period one corresponds to the period of indication extension and subsequent vaccination rollout to pediatric population following the approval for ages 12–15. https://www.ema.europa.eu/en/news/first-covid-19-vaccine-approved-children-aged-12-15-eu). All persons vaccinated were provided with clear instructions about which side effects to expect and how to report them to the VC. According to the available protocol, vaccinated people were advised to stay in the hospital, under close observation for 15 min after the administration of the vaccine. The critical comments are shortly outlined below and, where possible, patients' quotes have been used to vividly support the experiences gained vividly.

### First Period: Vaccination of the Elderly

(a) Physical function was limited in many of the presenting people and the majority needed assistance. Almost half of them were on wheelchairs, while the presence of frailty was quite apparent in several.
(b) Although almost all older people were living with or close to their children, they frequently expressed feeling isolated, potentially because of the measures taken by their children to protect their parents. One lady mentioned: “*It is very frustrating to live in the same building with my six grandchildren and not to be able to see them. I wish this vaccine will end this torture.”*
  GPs involved in the vaccination rollout set up a plan prior to its initiation so that, apart from vaccination, an empathetic and compassionate approach would be adopted independently of time limitations. If necessary, GPs would provide further consultations. Almost all elderly reacted positively to the empathetic approach of the healthcare professionals and expressed their gratitude for the warm and friendly services they were receiving. A man said: “*I wish there were no other people in the waiting room so I could stay more with you and discuss. I felt so lonely, and you are so welcoming…. I wish I knew where to find you when all this ends.”*
  Many people also shared stories from when they were younger and more active. A well-known retired medical doctor of 89 years who presented for vaccination said: “*I am glad that you made us feel so welcome; you remind me the days I was young, and I felt so close to my patients.”*
  The overall experience confirmed that time should be dedicated to really connecting with patients, even presents certain concrete challenges given the limitations in terms of time available for the encounters in the context of vaccine administration at the VC.
(c) Older people were almost always accompanied by their children, who were present during consultations. These caregivers asked a lot of questions regarding possible vaccination side effects. Discussions in the context of the encounters at the VC revealed that there might be plenty of room for interventions regarding their own vaccination coverage and several other issues related to disease prevention. This underlined the strong bonds between family members, —a valid characteristic of the Greek society, which has primarly contributed to counteracting the effects of previous crises- and highlighted the need to support family cohesion during the strenuous times of the pandemic.
d) The most challenging task was approaching older people with severe mental disorders and dementia, because of the inability to clearly communicate feelings and any potential side effects and given issues of addressing decision-making and autonomy. This challenge had to be managed through close contact with patient caregivers and family, to which GPs explained how to recognize and report side effects.

### Second Period: The First Negative Reactions and Vaccination Hesitancy

In the second period, an increased hesitancy to the vaccinations was reported, as captured in the Pharmacovigilance Risk Assessment Committee (PRAC) regarding the sporadic cases of unusual blood clots with low blood platelets with Vaxzevria (AstraZeneca's COVID-19 vaccine) (https://www.ema.europa.eu/en/news/meeting-highlights-pharmacovigilance-risk-assessment-committee-prac-6-9-april-2021). This resulted in a high level of anxiety and uncertainty to the local populations, affecting the vaccination rate, especially in many rural areas where these two vaccines were the only ones available. Furthermore, The lack of active and personal involvement of GPs and PHC professionals in communicating the explicit messages and information as the frontline physicians, with a sporadic role in the vaccination rollout, enhanced the hesitancy, and increased the existing confusion. Several proposals were put forth to local stakeholders and presented in the local media but were not implemented. Among them, the following have been mentioned:

- Establishing a telephone line to facilitate effective communication with people experiencing high fear and anxiety;
- Engaging local stakeholders and key local actors to communicate the key messages in a more effectively way, in addition to the centrally delivered by TV and the social media messages.

One major challenge that Greece faces is the fact that PHC is still nascent despite some progress in the past few years (2). Across the world, the role of PHC during this pandemic was considered crucial, representing the epicenter of service delivery (3). In Greece the pandemic revealed existing issues and this large gap, as people didn't have a “doctor” to trust and to discuss their concerns in case they needed more information or were hesitant (4).

### Third Period: Pediatric Populations

In the last period, the contact with children and families revealed the need for closer focus on the family and the adaptation of all the measures to the expectations, wishes, and needs of both the children and their parents, and for key messages to communicate soundly the benefits and soundly. Again, the need of primary care transformation and need for involvement of GP/FM was evident given how trust relationships can contribute to quality service delivery and outcomes.

## Discussion

The observations presented in this paper should be discussed in the light of efforts that current healthcare systems undertake to make health and social care services more resilient. It is a general agreement that strengthening PHC is the key to achieve it, while OECD and EC Expert Panel on Effective Ways of Investing in Health underlines the need to strength PHC with a focus on multidisciplinary teams, integrated with community health services, and equipped with digital technologies (5, 6).

Our second observation and especially the first quote, is also an indicates of the perception of what complete full vaccination signaled to them in terms of ending the pandemic.

An observation deserving further attention, especially when GP/FM meets older people with cognitive impairment, is the role of autonomy in the decision-making process, as well as the role of the GP/FM in reporting side effects, pharmacovigilance, and early safety signal identification. Specific recommendations regarding these aspects can be found in the framework of the European guidelines for good pharmacovigilance practices (GVP). Furthermore, safeguarding mobility and autonomy for these people in Greece may be an important area of focus. According to the 2019 OECD report “*In 2017, life expectancy at age 65 was 20.1 years, slightly higher than in EU countries. However, people in Greece can expect to live only about 40 % of these years without disability, compared to about 50 % in the EU, which translates into two healthy life years less”* (7). This highlights the need for public health and PHC measures to support this part of the population and their families. In addition, frailty has been proven to be a relatively good independent predictor/prognostic factor for COVID-19 outcomes, so this was an opportunity to map and inform on population-level characteristics (8, 9).

Equally, our observations point out to the impact of social isolation and the need for strong vital networking for people of this age group (over 85 years). In Greece, the role of family is crucial, and it seemed that, in our setting, there was good support provided to the elderly. However, loneliness was very prevalent and the need to see able to see their grandchildren was amongst the people's first wishes and perhaps a reason that motivated them to be vaccinated. We propose that vaccination programmes can be used for opportunistic screening of those elderly that need support in the form of mental and social care interventions, by easily identifying people without family or support/members or those that face other difficulties in accessing healthcare services. In line with the recommendations of the EC Expert Panel on effective ways to invest in health (10), the need to focus more on a comprehensive approach to old and vulnerable people including those who have a mental health condition and are socially and economically marginalized, by visiting them at home; assessing their health status, meeting their care and health needs seems to be an urgent priority; and current challenge for the Greek GP/FM and PHC.

To that direction, new healthcare models to respond to the current challenges should be discussed for example. The Italian healthcare system invested locally during the pandemic, and the idea is to keep patients near their families and friends who can take care of them (11). GP/FPs have the chance to work in teams with nurses and other specialists, including psychologists and dieticians to provide care close to patients. Although in Greece models based on such a decentralized form have still not been largely tested, in Greece, community-oriented care could potentially be one of the most successful stories shortly. GP/FM should take the lead toward the establishment of new organizational models.

In parallel, the role of compassion and empathy has received a prompt attention during the past years, and its impact has been assessed during the pandemic. A Lancet perspective article stated that “*Our empathy, our capacity to envision that we too could be affected, has been a powerful tool in the public health arsenal*” (12). However, this remains another challenge for the Greek healthcare system and its reform.

Health technology has been used in the fight against this pandemic; for example, remote consultations have been extensively used (13, 14). However, this is also a challenge as not all healthcare professionals have adequate digital literacy, whereas not everyone has access to new technologies. Furthermore, evaluation and assessment of these technologies to optimize their delivery and harvest their potential lags. Williams and Tsiligianni (3) report that health information and technology should follow the equity rules. They must be provided for the estimated 40% of the world not yet online. Health Technology Assessment International (HTAi) has also developed two position statements as a response to the COVID-19, emphasizing both the need of ensuring high evidentiary standards, and the need to have sound value propositions with early vaccine assessment for communication with the public (https://htai.org/hta-support-for-covid-19/position-statements-and-hta-reads).

The high hesitancy to COVID-19 vaccination observed during the second period calls for an effective communication strategy to recover from the pandemic, based on sound evidence and with aligned messages across jurisdictions, communities and, even, across borders. Such a strategy needs to involve and engage stakeholders and community leaders. PHC seems to be the suitable ground to achieve it and there are several positive examples gained from Crete that they can be used as best practices (15). To that direction, GP/FPs and PHC practitioners need to be equipped with skills of risk communication and to be able to provide appropriate management based on rational approaches and a clear understanding of people's risk perceptions (16–18). In addition, according to a recent publication (18), risk perception, motivation, and health literacy, all important predictors of health-seeking behavior and adherence to measures, needed to be addressed.

Finally, the vaccination to children and adolescents in the third period highlights the importance the role of the family-oriented PHC and underlines the needs for further training of GP/FPs in the field of child care.

## Conclusion

The COVID-19 pandemic has placed health systems under pressure and has led to the introduction of extraordinary, if necessary, measures in Greece and across the world. Older people and patients with pre-existing medical conditions and, in general, with vulnerability are at higher risk of infection and worse outcomes (19). Integrated care can help address the public health challenges brought to the fore by the pandemic, and several responses at the European level (20), including nested integrated care approaches, can be used by health systems toward this direction. Rethinking PHC and the care of older and vulnerable people within the public health context is known to be essential for non-communicable diseases (21) and our preliminary observations suggest that this also holds true for COVID-19. GP/FM should approach the persons and the families by taking into consideration their health's biological, environmental, social, and psychological determinants of their health.

Taking into consideration the critical stage of the pandemic and the PHC reforms lately unfolding in Greece (22), our experiences although they were based on personal observations from one VC, can provide ground for further research and support the efforts toward upgrading the country's PHC system toward its integration with public health and substantial involvement of PHC in the design and delivery of efficient and effective vaccination strategies (15). Strengthening PHC is the core message of a recent OECD report (5); Greece should urgently invest in community and family-oriented PHC as a key priority to ensure a resilient health system, increased preparedness and improved response. It could be considered as an important step toward strategic and evidence-informed planning to overcome hesitancy and improve the vaccination rollout in terms of efficiency and effectiveness, improving the overall uptake in across population groups in Greece (23).

## Author Contributions

All authors listed have made a substantial, direct, and intellectual contribution to the work and approved it for publication.

## Conflict of Interest

The authors declare that the research was conducted in the absence of any commercial or financial relationships that could be construed as a potential conflict of interest.

## Publisher's Note

All claims expressed in this article are solely those of the authors and do not necessarily represent those of their affiliated organizations, or those of the publisher, the editors and the reviewers. Any product that may be evaluated in this article, or claim that may be made by its manufacturer, is not guaranteed or endorsed by the publisher.

## References

[1] European Commission. European Commission Authorises First Safe Effective Vaccine Against COVID-19. (2020). Available online at: https://ec.europa.eu/commission/presscorner/detail/en/IP_20_2466 (accessed january 15, 2022).

[2] Lionis C Symvoulakis EK Markaki A Vardavas C Papadakaki M Daniilidou N. Integrated primary health care in Greece, a missing issue in the current health policy agenda: a systematic review. Int J Integr Care. (2009) 9:e88. 10.5334/ijic.322 19777112 PMC2748181

[3] Williams S Tsiligianni I. COVID-19 poses novel challenges for global primary care. NPJ Prim Care Respir Med. (2020) 30:30. 10.1038/s41533-020-0187-x 32555160 PMC7303134

[4] Gountas I Hillas G. Souliotis K.. Act early, save lives: managing COVID-19 in Greece. Public Health. (2020) 187:136–9. 10.1016/j.puhe.2020.08.016 32961466 PMC7447221

[5] OECD. Strengthening the Frontline: How Primary Health Care Helps Health Systems Adapt During the COVID 19 Pandemic. (2021). Available online at: https://www.oecd.org/coronavirus/policy-responses/strengthening-the-frontline-how-primary-health-care-helps-health-systems-adapt-during-the-covid-19-pandemic-9a5ae6da/ (accessed january 15, 2022).

[6] EC Expert Panel on Effective Ways of Investing in Health. The Organisation of Resilient Health and Social Care Following the COVID-19 Pandemic. Available online at: Panelhttps://ec.europa.eu/health/sites/default/files/expert_panel/docs/026_health_socialcare_covid19_en.pdf (accessed january 15, 2022).

[7] OECD. State of Health in the EU Greece Country Health Profile. Available online at: https://www.oecd.org/greece/greece-country-health-profile-2019-d87da56a-en.htm (accessed january 15, 2022).

[8] Zhang XM Jiao J Cao J Huo X-P Zhu C Wu XJ. Frailty as a predictor of mortality among patients with COVID-19: a systematic review and meta-analysis. BMC Geriatr. (2021) 21:186. 10.1186/s12877-021-02138-5 33731018 PMC7968577

[9] Hewitt J Carter B Vilches-Moraga A. The effect of frailty on survival in patients with COVID-19 (COPE): a multicentre, European, observational cohort study. Lancet Public Health. (2020) 5:e444–51. 10.1016/S2468-2667(20)30146-8 32619408 PMC7326416

[10] Barros P. Introduction to the Expert Panel on Effective ways of investing in health (EXPH) and its mandate. Eur J Public Health. (2021) 31(Suppl. 3):ckab164.147. 10.1093/eurpub/ckab164.147

[11] Politico. When Smaller Is Better: Italian Health Care Goes Local After Pandemic. (2021). Available online at: https://www.politico.eu/article/italy-health-care-hospital-local-coronavirus-pandemic/ (accessed january 15, 2022).

[12] Galea S. The art of medicine: compassion in a time of COVID-19. Lancet. (2020) 395:1897–8. 10.1016/S0140-6736(20)31202-2 32450108 PMC7255177

[13] van der Velden AW Bax EA Bongard E Munck Aabenhus R Anastasaki M Anthierens S. Primary care for patients with respiratory tract infection before and early on in the COVID-19 pandemic: an observational study in 16 European countries. BMJ Open. (2021) 11:e049257. 10.1136/bmjopen-2021-049257 34326052 PMC8326026

[14] Wanat M Hoste M Gobat N Anastasaki M Böhmer F Chlabicz S. Transformation of primary care during the COVID-19 pandemic: experiences of healthcare professionals in eight European countries. Br J Gen Pract. (2021) 71:e634–42. 10.3399/BJGP.2020.1112 33979303 PMC8274627

[15] Lionis C Petelos E Papadakis S Tsiligianni I Anastasaki M Angelaki A. Towards evidence-informed integration of public health and primary health care: experiences from Crete. Public Health Panorama. (2018) 4:699–713. Available online at: https://tiems.info/dmdocuments/events/TIEMS_2008_Bernd_Rohrmann_Keynote.pdf

[16] Rohrmann B. Risk perception, risk attitude, risk communication, risk management: a conceptual appraisal. In: Proceedings of the International Emergency Management Society Annual Conference. Prague (2008). Available online at: https://tiems.info/dmdocuments/events/TIEMS_2008_Bernd_Rohrmann_Keynote.pdf

[17] Rohrmann B. Renn O.. Risk perception research. An introduction. In: Cross-cultural Risk Perception. A Survey of Empirical Studies. Berlin: Springer(2000). p. 11–53.

[18] Priesemann V Balling R Bauer S Beutels P Valdez AC Cuschieri S. Towards a European strategy to address the COVID-19 pandemic. Lancet. (2021) 398:838–9. 10.1016/S0140-6736(21)01808-0 34384539 PMC8352491

[19] World Health Organization. Q&A on Coronaviruses (COVID-19). Available online at: https://www.who.int/news-room/q-a-detail/q-a-coronaviruses (accessed january 15, 2022).

[20] Lindner S Kubitschke L Lionis C Anastasaki M Kirchmayer U Giacomini S. Can Integrated Care Help in Meeting the Challenges Posed on Our Health Care Systems by COVID-19? Some Preliminary Lessons Learned from the European VIGOUR Project. Int J Integr Care. (2020) 20:4. 10.5334/ijic.5596 33132789 PMC7583206

[21] Tziraki C Grimes C Ventura F O'Caoimh R Santana S Zavagli V. Rethinking palliative care in a public health context: addressing the needs of persons with non-communicable chronic diseases. Prim Health Care Res Dev. (2020) 21:e32. 10.1017/S1463423620000328 32928334 PMC7503185

[22] World Health Organization. Greek Health Reform: Opening of New Primary Health Care Units. (2017). Available online at: http://www.euro.who.int/en/countries/greece/news/news/2017/12/greek-health-reform-opening-of-new-primary-health-care-units (accessed january 15, 2022).

[23] European Centre for Disease Prevention and Control Overview of the Implementation of COVID-19 Vaccination Strategies and Deployment Plans in the EU/EEA. Technical report (2021). Available online at: https://www.ecdc.europa.eu/en/publications-data/overview-implementation-covid-19-vaccination-strategies-and-deployment-plans (accessed january 15, 2022).

